# Management of extensive subcutaneous emphysema using negative pressure wound therapy dressings

**DOI:** 10.1002/rcr2.544

**Published:** 2020-02-27

**Authors:** Nai‐Chien Huan, Noorasyikin Mohamed Arifin, Teng‐Shin Khoo, Yean‐Chen Lai

**Affiliations:** ^1^ Department of Respiratory Medicine Queen Elizabeth Hospital Kota Kinabalu Malaysia; ^2^ Department of Medicine Labuan Hospital Labuan Malaysia

**Keywords:** Chest tube, negative pressure wound therapy, pneumothorax, subcutaneous emphysema, thoracic surgery

## Abstract

Subcutaneous emphysema (SE) is a common but usually self‐limiting complication of cardiothoracic procedures. Rarely, it can be life threatening and is characterized by extensive cutaneous tension and airway compromise requiring immediate intervention. There is a paucity of data on the most efficacious treatment methods for extensive SE. We report an 80‐year‐old gentleman who developed massive SE necessitating intubation for airway protection following a right chest tube insertion for spontaneous secondary pneumothorax. His SE persisted despite adequate thoracic drainage via a new chest tube. It was then decided to insert two negative pressure wound therapy dressings (NPWTD) or vacuum dressings in the patient's subcutaneous tissue layer via incisions made at anterior chest wall. The dressings were removed after four days in view of significant improvements. NPWTD appears to be an effective, well‐tolerated, safe, and inexpensive approach that hastens the resolution of SE without the need for invasive thoracic surgeries.

## Graphical abstract

To introduce negative pressure wound therapy dressings (NPWTD) as a cheap, effective, safe, and novel technique in patients with extensive subcutaneous emphysema (SE), we report an 80‐year‐old gentleman who developed extensive SE post chest tube insertion for secondary spontaneous pneumothorax necessitating intubation. After exhaustion of other methods, he underwent NPWTD via two subcutaneous incisions made at chest wall. He responded well to NPWTD and was extubated three days later.

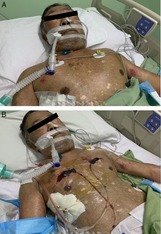

## Introduction

Subcutaneous emphysema (SE) is a common but usually self‐limiting complication of cardiothoracic procedures including thoracostomy. Rarely, it can be life threatening characterized by the presence of extensive cutaneous tension, palpebral closure, dysphagia together with pneumomediastinum, pneumoperitoneum, and airway compromise. Currently, there is a paucity of data on the most efficacious treatment methods for extensive SE, although various techniques such as negative pressure suction dressings, creation of “blowholes,” and insertion of angiocatheters have been described. Here, we report negative pressure wound therapy dressing (NPWTD) or vacuum dressing as an effective, safe, and cheap technique for management of extensive SE.

## Case Report

**Figure 1:**
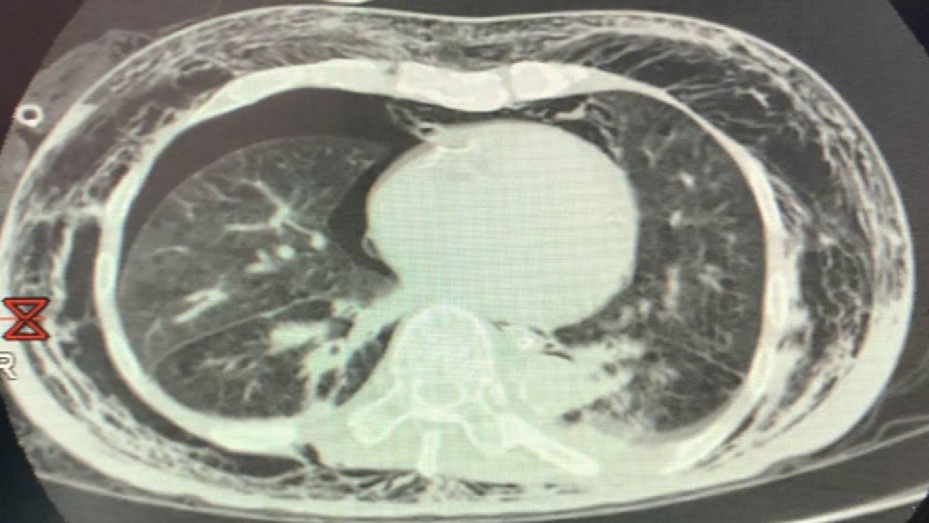
Computed tomography (CT) of the thorax demonstrating extensive subcutaneous emphysema together with right pneumothorax and pneumomediastinum.

**Figure 2:**
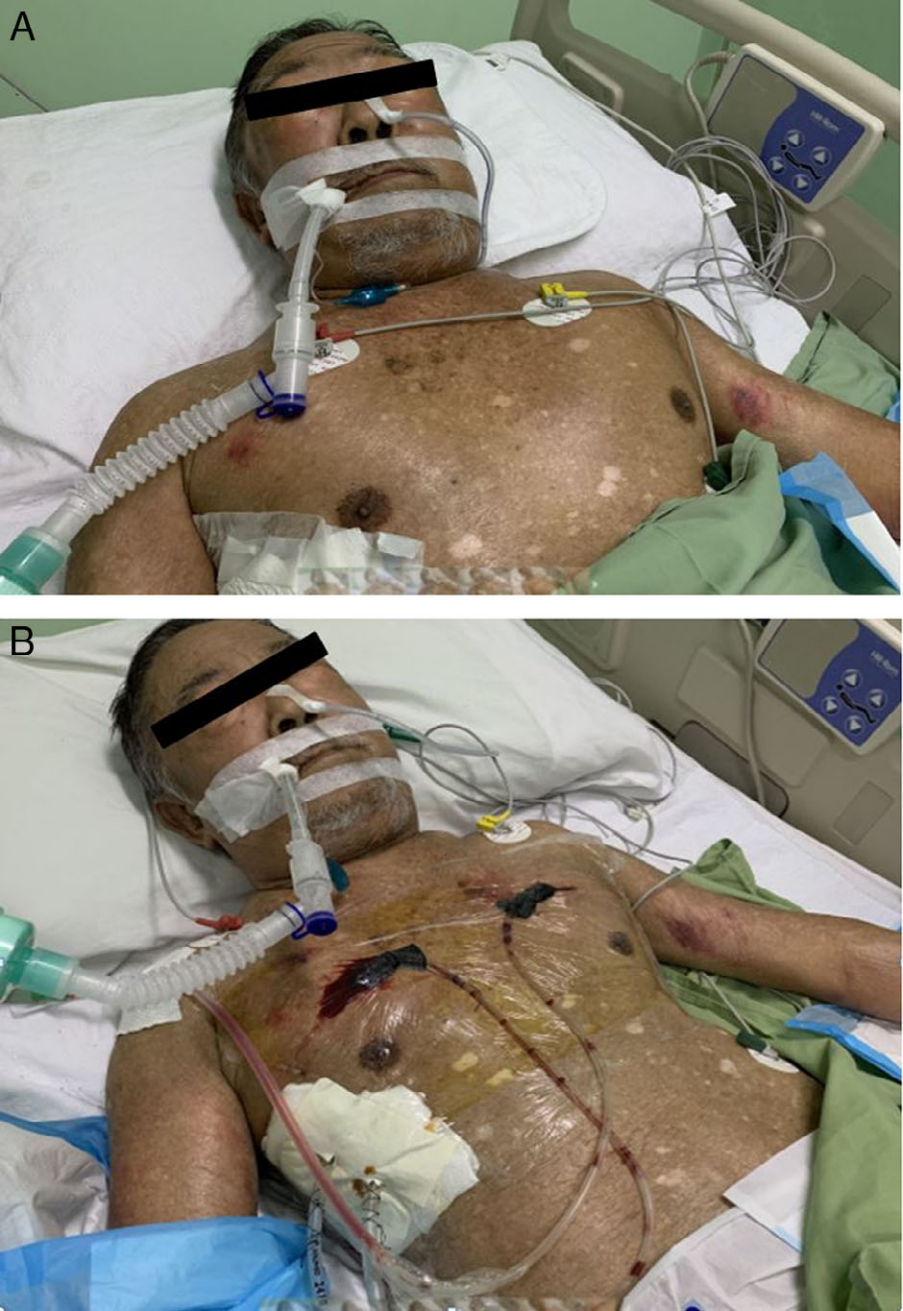
(A) Photograph demonstrating extensive subcutaneous emphysema post chest tube insertion extending from chest tube site to upper torso, neck, and face, leading to airway compromise. The patient was intubated for airway protection. (B) Photograph demonstrating improvement post negative pressure wound dressing inserted subcutaneously via incisions made at left and right anterior chest wall. Photograph taken approximately 24 h post insertion of wound dressings.

We report an 80‐year‐old gentleman with a background history of chronic obstructive pulmonary disease (Global Initiative for Chronic Obstructive Lung Disease (GOLD) Grade D; Modified Medical Research Council Dyspnoea Scale (mMRC) class III) who was admitted for a right spontaneous secondary pneumothorax. He unfortunately developed extensive SE one day after right chest tube insertion leading to hoarseness of voice, tense palpebral fissures, and tense neck swellings. Computed tomography (CT) of the thorax demonstrated extensive SE together with the presence of pneumomediastinum (Fig. 1). CT images also revealed that the tip of the chest tube was seen penetrating through part of his lung parenchyma which is the most likely cause of extensive SE. An urgent cardiothoracic surgery consult was made but unfortunately our patient was deemed unfit for surgery in view of his advanced underlying lung condition with poor lung reserve. He was intubated for airway protection on the fourth day of stay in view of development of stridor (Fig. 2A). Our patient continued to develop worsening SE despite adequate thoracic drainage via a new chest tube. Earlier attempts of subcutaneous cannula insertions for cutaneous tension release were not successful.

A multidisciplinary team discussion involving physicians, nurses, surgeons, physiotherapists, intensive care doctors, and patient's family members was conducted on the fifth day of hospitalization. It was then decided to insert two NPWTD in patient's subcutaneous tissue via incisions made at left and right anterior chest wall, 5 cm below the clavicle level. Extra precautions were made to ensure that the incisions were made at superior borders of the rib below and approximately 5 cm from the midline to avoid injuries to the intercostal and internal mammary vessels, respectively. The NPWTDs were created by using trimmed sterile sponge to fit the wound shape. The sponge was then wrapped around a modified nasogastric tube containing self‐created fenestrations on one end to aid in suction. Sutures were used to secure the sponge on the nasogastric tube. The other non‐fenestrated end of the nasogastric tube was connected onto a suction machine. After achieving wound haemostasis, the sponges were carefully inserted into the two subcutaneous incisions and secured by using sterile transparent drapes (Fig. 2B). Continuous negative pressure of 10 mmHg with sequential increment up to 50 mmHg was used.

Prompt regression of SE was noticed within the first 24 h followed by successful extubation after four days of NPWTD (Fig. 2B). External pressure and massage were done intermittently to enhance exit of trapped gas. In view of significant improvements, the drains were removed after four days from the date of insertion with no recurrence of SE following removal.

## Discussion

While most SE is self‐limiting, SE leading to circulatory compromise or airway collapse mandates prompt removal of trapped subcutaneous air. Surgery in the form of open thoracotomy or video‐assisted thoracoscopic surgery (VATS) is indicated but often not tolerated in critically ill patients with multiple comorbidities and poor lung reserves. As a result, various less invasive decompression methods have been described, including trochar chest tube insertions as subcutaneous drains 1, infraclavicular blow hole insertions 2, Jackson‐Pratt drains 3, Seldinger drains, and spiral‐fenestrated microcatheters 4, each with varying levels of success.

NPWTDs, whilst more established in its use for wound healing and reconstructive surgeries, are considered novel techniques in the management of extensive SE. Technical aspect of this technique is simple and straightforward: broad contact sponge with the surgically created wound in conjunction with high negative pressure suction to aid in removal of trapped subcutaneous air via patent tubes. Pain from suction and risks of wound infection are the two main concerns of this technique, which can be addressed by providing adequate analgesia (or sedation if patient is ventilated) and by frequent changing of sterile drapes respectively.

Our online PubMed search for “negative pressure wound therapy” and “subcutaneous emphysema” yielded only four relevant papers with a total number of 13 patients treated, albeit each with different technique and indications. Currently, there are no large‐scale studies or randomized controlled trials to compare efficacy and success rates of NPWTDs for SE. Whilst some described cases involved deep blowhole incisions up till the pectoral fascia 5, our patient was subjected to a less invasive procedure, with only breach of the subcutaneous layer. Our technique involved only the simplest of tools and can therefore be employed effectively, even in resource‐limited settings. In light of our positive experience, we recommend NPWTD to be considered in patients with extensive SE who are unfit for surgery and are not responding to other less invasive measures such as chest tube suctioning and insertion of subcutaneous cannulas to aid in gas release. Paucity of published evidence is the main limitation for stronger recommendation of this technique.

In the absence of large‐scale studies on identifying the most effective measure to treat massive SE, NPWTD appears to be an effective, safe, and cheap approach that hastens the resolution of SE without the need for more invasive thoracic operations.

### Disclosure Statement

Appropriate written informed consent was obtained for publication of this case report and accompanying images.
